# An Approach to Re-evaluate the Reference Cutoff of the Parameters of Newborn Screening: An Observational Study

**DOI:** 10.7759/cureus.45139

**Published:** 2023-09-12

**Authors:** Suprava Patel, Neharani Verma, Phalguni Padhi, Seema Shah, Rachita Nanda, Eli Mohapatra

**Affiliations:** 1 Biochemistry, All India Institute of Medical Sciences, Raipur, Raipur, IND; 2 Neonatology, All India Institute of Medical Sciences, Raipur, Raipur, IND

**Keywords:** multiple of median, percentile, reference range, term healthy newborn, nbs

## Abstract

Background

Unless a cutoff level of the parameters of newborn screening (NBS) is defined, a screening test's results would end in high recall rates and apprehensive parents. The study aimed to establish a cutoff level of the healthy term newborns.

Materials and methods

The study was a retrospective observational data analysis on a cohort of 1158 term newborns who underwent NBS in our institute. The percentile distribution of the NBS parameters was computed and the 99th percentile value was considered the new cutoff. For lower values, such as neonatal glucose 6-phosphate dehydrogenase (nG6PD) and neonatal biotinidase (nBIOT), low percentile values were regarded as new cutoff value.

Results

Neonatal thyroid stimulating hormone (nTSH), nG6PD, neonatal immunoreactive trypsinogen (nIRT), and nBIOT showed a wide variation in the distribution. Most newborns had neonatal galactose (nGAL), nIRT, and nBIOT values above the median. The 99th percentile value of nTSH was 14.5 mIU/L, and that of neonatal 17-hydroxyprogesterone (n17-OHP) was 43.7 nmol/L. The 1.0th percentile value for nG6PD was decreased to 2.18 IU/gHb. The new cutoff values for nBIOT, nIRT, neonatal phenylketonuria (nPKU) and nGAL were 48.59 U, 95.3 µg/L, 2.3 mg/dL and 15.9 mg/dL. The mean and median nTSH values did not significantly differ (p=0.99) in the first five days of birth. On the contrary, the study population depicted considerably raised levels of n17-OHP on day 3, followed by a sharp decrease (p=0.029). Similarly, nIRT displayed significant differences in the first five days (p=0.017).

Conclusion

Using the 99th percentile values of the NBS parameters as the new cutoff levels might be beneficial in terms of the recall rates and cost burden.

## Introduction

Newborn screening (NBS) is the most effective method for identifying a specific group of inherited and metabolic disorders in infants. It is not meant to establish a diagnosis, but abnormal parameters are intended to assess the risk for a specific disorder. For a confirmatory diagnosis, the positively screened newborns are advised for additional investigations for the suspected condition. In newborns with mild phenotypes or absence of a classical form of the disorder, the values might not vary much from the biological reference interval in newborns with no diseases in the true sense [1]. Therefore, unless an algorithm is established and a cutoff level of the parameters is defined, a screening test's positive predictive value would remain low, impacting the health system with high false-positive results and apprehension among parents. To date, there are no defined cutoff levels of the biochemical parameters analyzed in the dried blood spot (DBS) cards [2]. The cutoff may be a decreased analyte level or an elevated level due to the accumulation of the analyte. However, reference ranges and the cutoff levels of individual analytes vary based on the analytical method and the instrument used to perform the assays in each laboratory [3]. The reference values are never universal and should be evaluated for each demographic population [4]. However, due to resource constraints, this process is quite complicated for a single laboratory to establish a reference cutoff using high-standard analytical techniques like liquid chromatography mass spectrometry (LC/MS-MS). The limitations are further constrained in NBS programs in developing countries like India, where parents disagree with a prick due to a lack of awareness, especially when the newborn seems healthy during the first week. Appropriate clinical data management can also be considered to obtain the reference values provided stringent analytical and preanalytical factors have been maintained in the laboratory [5].

Considering the above facts, the study aimed to establish a cutoff level of the NBS parameters processed through the first-tier immunoassay method in apparently healthy term newborns.

## Materials and methods

The study was a retrospective observational data analysis on the cohort of term newborns (delivered at 37 to 42 weeks of gestation) who underwent NBS in our institute. The samples were collected by heel prick on Whatman 903 filter paper per the standard protocol [6]. The DBS samples were processed only after qualifying the preanalytical quality check, such as sample collection, storage, and transport, as per the Clinical and Laboratory Standards Institute (CLSI) document NBS01-A6 [6]. The NBS parameters were processed by enzyme activity by immunofluorescence method-based neonatal kits by Labsystems Diagnostics (Vantaa, Finland) as listed in Table 1.

**Table 1:** The list of clinical manifestations in the children for diagnosis or suspicious for the specific disorders nTSH - neonatal thyroid stimulating hormone; nG6PD - neonatal glucose 6-phosphate dehydrogenase; nCAH - neonatal congenital adrenal hyperplasia; n17-OHP - neonatal 17-hydroxyprogesterone; nBIOT - neonatal biotinidase; nPKU - neonatal phenylketonuria; nIRT - neonatal immuno-reactive trypsinogen; nGAL - neonatal total galactose.

Disorder	NBS Parameter	Clinical manifestation
Congenital hypothyroidism	nTSH	Loss of feeding, Constipation, Lethargy, Hoarse cry, Prolonged jaundice, Coarse facies, Large fontanelles, Umbilical hernia, Delayed milestone, Low IQ
G6PD deficiency	nG6PD	Anemia, Severe lethargy, Dark colored urine, Frequent jaundice, Jaundice after intake of few drugs like antibiotics, antimalarial, after eating some foods
Congenital adrenal hyperplasia (CAH)	n17-OHP	Crisis in classic severe salt-wasting form such as Poor feeding, Persistent vomiting, Loose stool/Diarrhea, Weak feeble cry, Failure to thrive, Dehydration, Lethargy, Hyponatremia, Learning disability, Ambiguous genitalia (in females)
Biotinidase deficiency	nBIOT	Cutaneous manifestations like Seborrheic dermatitis, Atopic dermatitis, Alopecia – complete/partial Neurological manifestations like Myoclonic seizures, Hypotonia, Sensory loss, Hearing loss
PKU	nPKU	Typical musty odor, Seizures, Skin rashes, Low skin pigmentation, Microcephaly, Intellectual disability, Delayed milestone, Behavioral and emotional issues, Mental health disorders
Cystic fibrosis	nIRT	Meconium ileus, Malnutrition, Poor growth, Frequent respiratory infection, Breathing difficulties, Lung damage, Nasal polyp, Pneumothorax, Rectal prolapse, Hemoptysis, Abdominal pain/inflammation in pancreas, Chronic diarrhea
Galactosemia	nGAL	Cataract, Mental retardation, Poor health, Hepatomegaly

The laboratory is enrolled for proficiency testing in samples received from the Centers for Disease Control (CDC), United States, under the Newborn Screening Quality Assurance Program (NSQAP) and the performance is satisfactory. As per the recommendation published by the American College of Medical Genetics and Genomic (ACMG) and Advisory Committee on Heritable Disorders in Newborns and Children (ACHDNC), all newborns must be screened for metabolic and inherited disorders as early as possible, and all positively screened newborns should be sent for confirmation via LC/MS-MS [7]. As a routine protocol, our laboratory also complied with the guideline. All newborns tested for NBS and found to have a positive screening result were recalled for a repeat testing of DBS by immunoassay method except for nTSH and G6PD. To confirm congenital hypothyroidism (CH), serum TSH and T4 levels were measured using Advia Centaur XP's chemiluminescence method. Glucose 6-phosphate dehydrogenase deficiency was confirmed by quantitative estimation of G6PD activity in whole blood using the enzyme kinetic method by G-Six kit from Tulip Diagnostics (Nagpur, Maharashtra, India). For all other parameters, if found positive for the second result, the parent was counseled for being tested for confirmatory testing by LC/MS-MS in another laboratory as our institute did not have the facility.

Of all the 2557 term newborns screened through NBS in our institute from 2018-2021, we sorted out those who weighed ≥2500 gm. The samples were collected within five days of birth; the newborns, who had no abnormal clinical presentations before discharge, had Apgar score at 1 and 5 minutes ≥7, breastfed, and discharged within five days of birth, were enrolled. Each newborn was followed for one year for developmental and clinical details. Those who could not be contacted gave a history of delayed milestones, or were diagnosed or suspected of specific disorders, including hemoglobinopathy, or ambiguous response, as delineated in Table 1, were excluded from the analysis. The NBS parameters of babies found clinically healthy during enrolment were considered for the final data computation to determine the reference range. A total of 1158 babies were finally considered for the data analysis. The newborns with positive results or clinical suspicion, or other factors that influence NBS results are not discussed in this article to avoid confusion and perplexity for the readers. This article focuses on the reference cutoff, and details of the other determinants shall be covered in another report.

Statistical analysis

The statistical analyses were performed using Microsoft Excel and IBM SPSS 26 (IBM Corp., Armonk, NY, USA). The mean, median, standard deviation (SD), standard error of the mean (SE), interquartile range (IOR), the minimum and maximum values were computed for all the NBS analytes. The distribution pattern of the analytes in the study population was illustrated using box plots after excluding the outliers. The percentile distribution of the NBS parameters were computed for 3rd, 5th, 10th, 25th, 50th, 75th, 90th, 95th, 97th, and 99th percentiles [8]. Low values are considered screening positive for the parameters such as nG6PD and nBIOT. For these two parameters, lower percentiles, 0.25th, 0.5th, 0.75th, 1.0th, 1.5th, 2.0th, 2.5th, 3.0th were calculated and 1.0th percentile value was considered as cutoff [8]. As per the International Federation of Clinical Chemistry (IFCC) recommendation, the reference interval of an analyte includes the 2.5th to 97.5th percentile of the apparently healthy population [9]. However, as NBS is a screening program to identify the disorder at an early age, the 99th percentile was set as the new cutoff for the parameters except for nG6PD and nBIOT (lower than cutoff values considered as screening positive), for which the 1.0th percentile value was considered the new cutoff. A higher cutoff was set with the intention of reducing recall rates and was considered to be more cost-effective [8,10]. The values mentioned in the kit brochures were considered the initial cutoff value appropriately validated during method standardization. To observe the difference in values of the parameters in the first five days, the newborns were grouped based on the days from birth as 1st, 2nd, 3rd, 4th, and 5th day of birth. The median values were compared using the Kruskal-Wallis test. The mean values were compared using ANOVA after the logarithmic transformation of the values normalized the data.

Few laboratories also prefer a floating cutoff for NBS parameters. These unitless cutoff values were calculated by dividing the patient analyte value by the median value obtained from the study population. The value was reported as a multiple of median (MoM).

## Results

The mean (SD), median, range, and initial cutoff values of the NBS parameters are depicted in Table 2. The nTSH ranged from 0.1 to 93.6 mIU/L in the study population, with a mean of 3.5 mIU/L and a median value of 3.1 mIU/L. The nG6PD ranged from 0.1 to 12.4 IU/gHb, with a mean of 9.23 IU/gHb and a median of 9.7 IU/gHb. The maximum value of n17-OHP observed was 216 nmol/L, whereas the mean and median values were 20.84 and 20.09 nmol/L, respectively. The IQR for nBIOT was 130.38 U (7.2-392.9). The mean value observed was 191.45, and the median was 176.35 U. Similarly, the highest nIRT level was 341.9 µg/L, while 25.52 and 21.2 µg/L were respectively the means and median values. The maximum nGAL depicted by the study population was 66.2 mg/dL, with a mean value of 2.49 mg/dL and a median of 1.3 mg/dL. The mean and median of nPKU were almost similar, 1.2 mg/dL, with an IQR of 0.6.

**Table 2:** The values of the NBS parameters in the study population $ denotes initial cutoff as per the kit brochure; ^ denotes the new cutoff as per the 99th percentile value calculated in this study nTSH - neonatal thyroid stimulating hormone; nG6PD - neonatal glucose 6-phosphate dehydrogenase; nCAH - neonatal congenital adrenal hyperplasia; n17-OHP - neonatal 17-hydroxyprogesterone; nBIOT - neonatal biotinidase; nPKU - neonatal phenylketonuria; nIRT - neonatal immuno-reactive trypsinogen; nGAL - neonatal total galactose.

NBS Parameters	Mean	SE	Median	IQR	Minimum	Maximum	Initial cutoff^$^	New cutoff^
nTSH (mIU/L)	3.5	3.8	3.1	2.3	0.1	93.6	<10	<14.5
nG6PD (IU/gHb)	9.23	2.01	9.7	2.7	0.1	12.45	>3	>2.18
n17-OHP (nmol/L)	20.84	10.2	20.09	11.15	0.4	216	<35	<43.7
nBIOT (U)	191.45	84.8	176.35	130.38	7.2	392.9	≥50	≥48.59
nPKU (mg/dL)	1.23	0.4	1.2	0.6	0.1	3.11	<2.5	<2.3
nIRT (µg/L)	26.52	21.7	21.2	22.13	0.1	341.9	<70	<95.3
nGAL (mg/dL)	2.49	4.2	1.3	3.2	0.1	66.2	<17	<15.9

**Figure 1:**
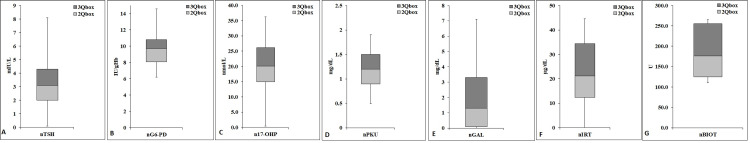
Box-plot presentation of the distribution of the NBS parameters in the study population NBS - newborn screening; nTSH - neonatal thyroid stimulating hormone; nG6PD - neonatal glucose 6-phosphate dehydrogenase; n17-OHP - neonatal 17-hydroxyprogesterone; nPKU - neonatal phenylketonuria; nGAL - neonatal total galactose; nIRT - neonatal immuno-reactive trypsinogen; nBIOT - neonatal biotinidase. Figure 1A denotes the median and the distribution of nTSH in mIU/L; Figure 1B denotes the median and the distribution of nG6PD in IU/gHb; Figure 1C denotes the median and the distribution of n17-OHP in nmol/L; Figure 1D denotes the median and the distribution of nPKU in mg/dL; Figure 1E denotes the median and the distribution of nGAL in mg/dL; Figure 1F denotes the median and the distribution of nIRT in µg/L; Figure 1G denotes the median and the distribution of nBIOT in U.

The box-plot representation of the distribution of the NBS parameters in the study population is illustrated in Figure 1. Although the range is wide, the nTSH showed a narrow IQR with a uniform distribution of the values (Figure 1A). nG6PD values depicted a slightly negatively skewed distribution (Figure 1B). A greater number of neonates had G6PD values below the median (9.7 IU/gHb). The distribution pattern for n17-OHP was wider compared to nTSH with a nearly symmetric distribution (Figure 1C). The nPKU values reported a very narrow and uniform distribution among the neonates of the study population (Figure 1D). The neonates recorded positive skewness for nGAL, nIRT, and nBIOT values indicating that most of them reported higher values (Figure 1E-1G). nIRT revealed a very wide distribution (Figure 1F) while nBIOT did not show a wider range (Figure 1G).

**Figure 2:**
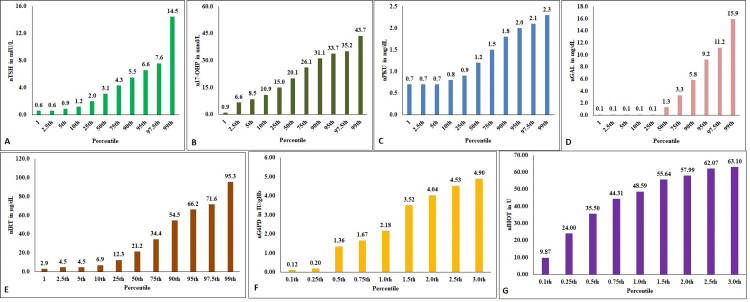
The mean values of the NBS parameters based on percentiles NBS - newborn screening; nTSH - neonatal thyroid stimulating hormone; nG6PD - neonatal glucose 6-phosphate dehydrogenase; nBIOT - neonatal biotinidase; n17-OHP - neonatal 17-hydroxyprogesterone; nPKU - neonatal phenylketonuria; nGAL - neonatal total galactose; nIRT - neonatal immuno-reactive trypsinogen. Figure 2A denotes the percentile distribution of nTSH values; Figure 2B denotes the percentile distribution of n17-OHP values; Figure 2C denotes the percentile distribution of nPKU values; Figure 2D denotes the percentile distribution of nGAL values; Figure 2E denotes the percentile distribution of nIRT values; Figure 2F denotes the percentile distribution of nG6PD values; Figure 2G denotes the percentile distribution of nBIOT values.

The percentile distribution of the mean values of the NBS parameters is illustrated in Figure 2. As reflected in Figure 2A, the 99th percentile values of nTSH were below 14.5 mIU/L, and the 97th percentile was 7.6 mIU/L. Similarly, 99th percentiles of the newborns, the values were below 43.7 nmol/L, 2.3 mg/dL, 15.9 mg/dL, and 95.3 µg/L of n17-OHP (Figure 2D), nPKU (Figure 2E), nGAL (Figure 2F), and nIRT (Figure 2G), respectively. Lower percentiles were calculated for the parameters like nG6PD and nBIOT, and the 1.0th percentile value was considered the cutoff. The 1.0th percentile values were 2.18 IU/gHb (Figure 2B) and 48.59 U (Figure 2C).

**Figure 3:**
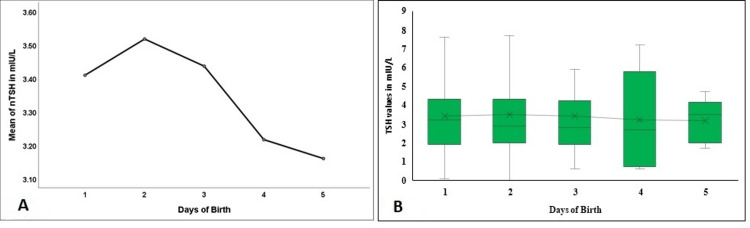
Comparison of the nTSH in the first five days of birth in the study population nTSH - neonatal thyroid stimulating hormone; Figure 3A denotes a comparison of the mean values in the first five days, Figure 3B denotes the comparison and the distribution of the nTSH levels in the first five days of birth.

**Figure 4:**
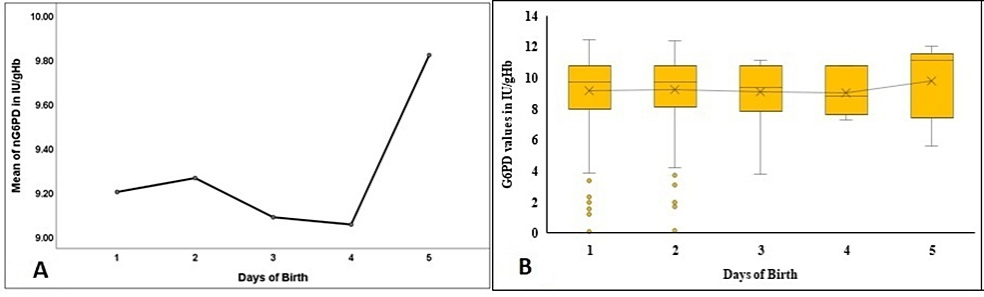
Comparison of the nG6PD in the first five days of birth in the study population nG6PD - neonatal glucose 6-phosphate dehydrogenase; Figure 4A denotes a comparison of the mean values in the first five days, and Figure 4B denotes the comparison and the distribution of the nG6PD levels in the first five days of birth.

**Figure 5:**
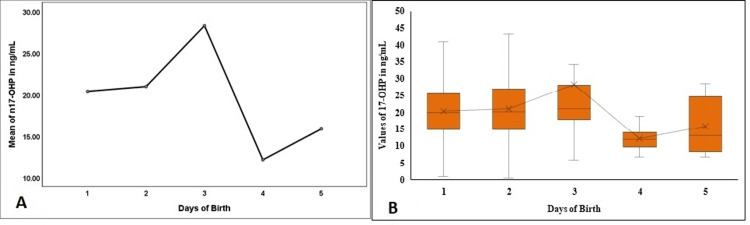
Comparison of the n17-OHP in the first five days of birth in the study population n17-OHP - neonatal 17-hydroxyprogesterone; Figure 5A denotes a comparison of the mean values in the first five days, and Figure 5B denotes the comparison and the distribution of the n17-OHP levels in the first five days of birth.

**Figure 6:**
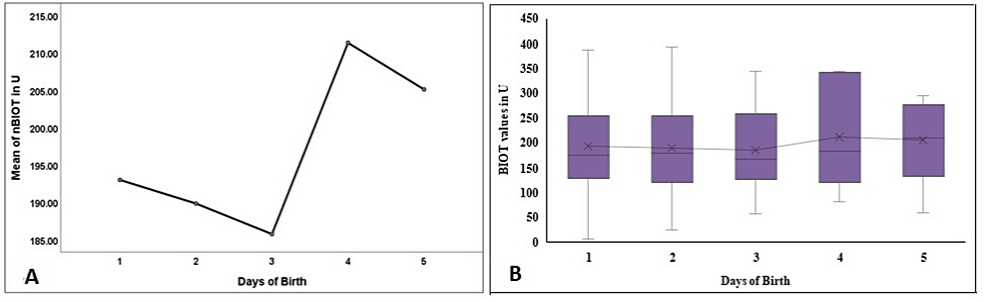
Comparison of the nBIOT in the first five days of birth in the study population nBIOT - neonatal biotinidase; Figure 6A denotes a comparison of the mean values in the first five days, and Figure 6B denotes the comparison and the distribution of the nBIOT levels in the first five days of birth.

**Figure 7:**
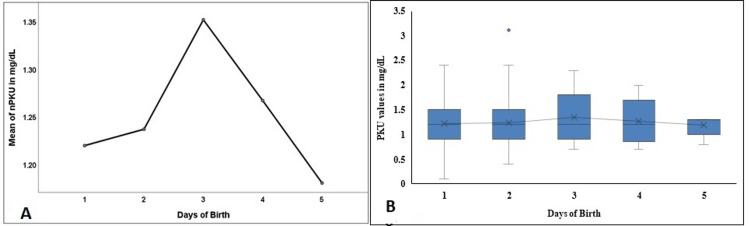
Comparison of the nPKU in the first five days of birth in the study population nPKU - neonatal phenylketonuria; Figure 7A denotes a comparison of the mean values in the first five days, and Figure 7B denotes the comparison and the distribution of the nPKU levels in the first five days of birth.

**Figure 8:**
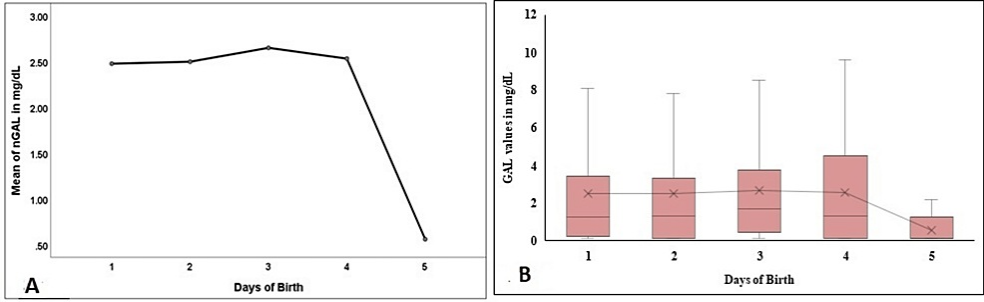
Comparison of the nGAL in the first five days of birth in the study population nGAL - neonatal total galactose; Figure 8A denotes a comparison of the mean values in the first five days, and Figure 8B denotes the comparison and the distribution of the nGAL levels in the first five days of birth.

**Figure 9:**
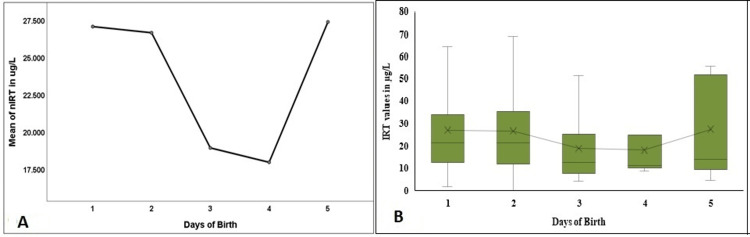
Comparison of the nIRT in the first five days of birth in the study population nIRT - neonatal immuno-reactive trypsinogen; Figure 9A denotes a comparison of the mean values in the first five days, and Figure 9B denotes the comparison and the distribution of the nIRT levels in the first five days of birth.

A comparison of the NBS parameters in the first five days of the birth of the newborn is elaborated in Figures 3-9. As depicted in Figure 3A, the mean nTSH did not show a significant difference (p=0.99), although a rising trend in the first two days, followed by a gradual fall by the fifth day of birth, was observed. Similarly, the median nTSH in Figure 3B did not differ significantly (p=0.274). The trend reflected that nTSH normalizes by the fifth day of birth.

Similarly, the mean (Figure 4A) and median nG6PD (Figure 4B) values did not differ in the neonates in the first five days (p=0.92).

As shown in Figure 5A, the mean n17-OHP levels significantly differed among the groups (p<0.001). The study population depicted considerably raised levels of n17-OHP on day 3, followed by a sharp decrease. The median values also recorded a similar trend in the newborns (p=0.029) (Figure 5B).

nBIOT, nPKU, and nGAL levels did not vary significantly in the first five days. However, a sharp dip on day 5 was observed in nGAL values (Figure 6A, 6B; Figure 7A, 7B; Figure 8A, 8B).

A significant variation was observed for median nIRT in the newborns' first five days of life (p=0.017). Third-day nIRT median was significantly lower than the first- and second-day values (Figure 9B). The mean nIRT levels did not vary significantly (p=0.18, Figure 9A).

**Figure 10:**
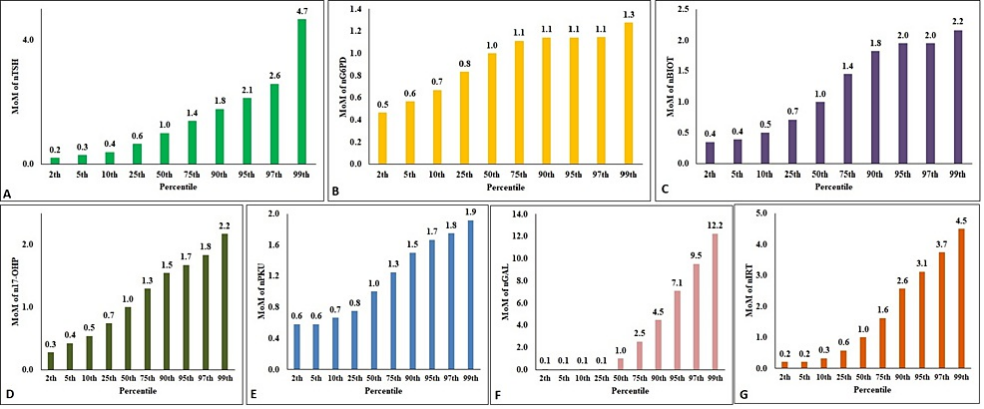
The Multiple of the Median values of the NBS parameters based on percentiles MoM - Multiple of the Medians; NBS - newborn screening; nTSH - neonatal thyroid stimulating hormone; nG6PD - neonatal glucose 6-phosphate dehydrogenase; n17-OHP - neonatal 17-hydroxyprogesterone; nBIOT - neonatal biotinidase; nPKU - neonatal phenylketonuria; nIRT - neonatal immuno-reactive trypsinogen; nGAL - neonatal total galactose. Figure 10A denotes the percentile distribution of nTSH MoM; Figure 10B denotes the percentile distribution of nG6PD MoM; Figure 10C denotes the percentile distribution of nBIOT MoM; Figure 10D denotes the percentile distribution of n17-OHP MoM; Figure 10E denotes the percentile distribution of nPKU MoM; Figure 10F denotes the percentile distribution of nGAL MoM; Figure 10G denotes the percentile distribution of nIRT MoM.

The MoM values were calculated per the percentiles used for the newborns' NBS parameters and illustrated in Figure 10A-10G. The 50th percentile value was 1.0 for all parameters. The 99th percentile MoM values for nTSH, nG6PD, n17-OHP, nBIOT, nPKU, nIRT, and nGAL were, respectively, 4.67, 1.28, 2.175, 2.17, 1.92, 4.497, and 12.25. The MoM of 17-OHP revealed a significant increase on 3rd day compared to other days (p<0.001). No significant differences were observed for the MoM of other NBS parameters when compared among the groups as per the days of birth.

## Discussion

NBS results in 1158 newborns were reviewed as they were considered apparently healthy after one year of age (as per the inclusion criteria) with no clinical signs and symptoms at the time of clinical documentation for the study. The 99th percentile cutoff value of 14.5 mIU/L for nTSH (Table 2 and Figure 2A) corroborated with the cutoff level reported by Khan et al. study as 15 µU/mL (15 µU/mL=15 mIU/L) for nTSH [8]. Verma et al. study observed a 99.5th percentile nTSH value as <20 mIU/L. Lowering the cutoff nTSH to 10 mIU/L led to an increase in recall rate by 0.1% to 2%, thus suggesting 20 mIU/L as the cutoff of nTSH in capillary blood specimens collected in DBS [11]. The cutoff values of nTSH across the globe varied widely from 6 mIU/L in Wales (Australia) to 30 mIU/L in Turkey [12,13]. The wide variations reported could be attributed to the differences in the assay technique, the differences in the age of the study population, or the type of sample used. Ontario NBS program reported that 24% of the newborns diagnosed with congenital hypothyroidism (CH) had nTSH values within 17-29.9 mIU/L [14]. The Italian screening program also mentioned that nearly 22% of newborns could be diagnosed with CH due to a lowered nTSH cutoff [15]. Other countries such as China, Sri Lanka, and Iran also recommended capillary nTSH >20 mIU/L as an indicator for immediate re-evaluation for CH [16-18]. Gopalakrishnan et al. study on age-related cutoffs documented a nTSH value of >34 mIU/L as the cutoff during 24-48 hours of birth and >20 mIU/L after 48 hours. The study showed a decreasing trend of the mean (SD) value of nTSH in newborns with an increasing number of hours of birth. The mean (SD) value of nTSH in newborns of less than 48 hours was 7.2 (5.2); for 48-72 hours was 6.1 (5.1), and ≥72 hours was 5.6 (4.8) mIU/L [19]. The present study also depicted a decreasing trend in the first five days of birth (Figure 3A, 3B). Di Dalmazi et al. study also denoted a similar trend in the first seven days of birth [20]. The higher levels of nTSH on the first and second days could be ascribed to the physiological neonatal surge for the hormone TSH followed by gradual settling of the hypothalamic-pituitary-thyroid axis after 72 hours of birth [13]. Accordingly, the American Academy of Pediatrics (AAP) and American Congress of Obstetricians and Gynaecologists (ACOG) suggested sampling for NBS after 48 to 72 hours of birth so that the TSH surge would subside. However, it might be a significant concern in developing countries like India with early discharge policies [21].

The cutoff level for nGAL used in the Gopalakrishnan et al. study was 6.5 mg/dL for which the recall rate was 4.1%. It was significantly reduced when the cutoff value was set to 11.7 mg/dL [19]. The cutoff value specified in the present study at the 99th percentile was 15.9 mg/dL (Figure 2D). However, the 11.7 mg/dL cutoff value set by the Gopalakrishnan et al. study was represented by the 97th percentile nGAL value of 11.2 mg/dL in the present study population. Similarly, the recall rate dramatically decreased for nBIOT after revisiting the cutoff to ≥45 U from ≥77 [19]. The cutoff value at the 1.0 percentile was 48.9 U (Figure 2G) which was entirely in agreement with the Gopalakrishnan et al. study and Khan et al. study that observed a 1.0% value of 49 U/dl [8,19].

A study by Anandi and Shaila reported the mean (SD) of n17-OHP 5.486 (3.96) ng/mL (16.601 nmol/L) in the study population and the cutoff value used was <9.6 ng/mL (<29.05 nmol/L). The mean (SD) of n17-OHP in term babies was 4.86 (2.47) (14.707 nmol/L), and the reported median value was 4.5 ng/mL (13.617 nmol/L) [22]. On the contrary, the initial cutoff value used in our study was <35 nmol/L (<11.566 ng/mL). The mean (SE) and median values depicted in the present study were 20.84 (10.2) and 20.09 nmol/L (Table 2). The differences in the values could be due to the difference in the analyte's analysis method. The 99th percentile value of 43.7 nmol/L (Figure 2B) depicted in the present study was equivalent to the 99th percentile value of n17-OHP, 44 nmol/L, reported by Khan et al. study [8]. The n17-OHP values in the present study were higher in the first 72 hours of birth, followed by a sudden decrease (p<0.001, Figure 5A, 5B). Studies have depicted that n17-OHP at birth or within 72 hours of delivery is usually high and gradually reduces in the next few days of life. Therefore, false positive rates are higher when collected within 72 hours of birth [23,24]. Although false positive rates are higher, sampling within three days of birth is recommended as early diagnosis's advantage outweighs the false positive rate's disadvantage [21,24].

Pitt's study considered 150 µmol/L (2.48 mg/dL) as the cutoff for nPKU. The 99th percentile value for nPKU depicted in the present study was 2.3 mg/dL (Figure 2C) [1].

Fu et al. determined a cutoff of 2.2 U/gHb for male newborns. For female newborns, <2.8 U/gHb was defined to be borderline and <1.6 U/gHb as deficient [25]. Likewise, Miao et al. study obtained a cutoff value of 2.35 and 2.55 U/gHb for male and female newborns, respectively, whereas 2.2 U/gHb was the estimated cutoff for G6PD by Kaur et al. [9,21]. Pan et al. study on 82,233 newborns referred to 2.35 U/gHb as the cutoff value for male and 3.65 U/gHb for female newborns [26]. The cutoff value computed in our study population at 1.0th percentile was 2.18 U/gHb (Figure 2F), equivalent to the cutoff levels reported by Kaur et al. study and close enough to Fu et al. and Miao et al. study.

Sadik et al. study fixed the 99th percentile value of 61 ng/mL (1 ng/mL = 1 µg/L) as the cutoff limit for nIRT [10]. Arrudi-Moreno et al. study on 790 newborns positive for CF estimated a cutoff reference as 76.2 ng/mL [27]. The mean nIRT value was 75.66 (median: 70.12; range: 60-270) ng/mL in healthy full-term newborns, whereas 175.82 (66-368) ng/mL for the CF confirmed cases. The nIRT levels tend to decrease after the third week of birth; thus, the cutoff point might vary according to the time of sample collection. The mean and median nIRT observed in our study population, 26.52 and 21.2 (range: 0.1-341.9) µg/L, were comparatively lower, and the new 99th percentile value for cutoff, 95.3 µg/L, was higher (Figure 2E). On the contrary, Kharrazi et al. suggested a lower cutoff of 40 ng/mL to reduce the number of false negative results for CF. The median nIRT was 39 ng/mL for all 61 CF false negative cases. The percentage for missed CF in nIRT cutoff level was 2.54% for the 96th percentile value and 4.55% for the 99th percentile value [28]. Nearly 80th percentile nIRT in the newborns in the present study showed a mean value of 40 µg/L. The differences could be due to the variation in the immunoassay principle or ethnic variation, or other factors that need to be looked into with a more robust study design. Besides, the nIRT values change remarkably with the days of birth, demographic profile, exposure and storage environment, the season of birth, and many more [10,27,28]. Hence, the cutoff value for nIRT needs to be further verified. Reporting MoM value along with the observed value might be another appropriate solution to be more precise [3].

The NBS parameters in a study population usually do not follow a Gaussian curve. The values vary a lot within a particular range. Few laboratories, therefore, might prefer reporting in MoM for the parameters that show quite a variation on a day-to-day basis. The variations observed in MoM values in a study population are minimal and closer to a Gaussian distribution (Figure 10). To be more precise, these values might be used in association with the fixed cutoff value while reporting [3].

Limitations and strength

The study's primary limitation is that we have analyzed the data retrospectively. Clinically healthy babies were included in the analysis. LC/MS-MS confirmations were unavailable in many neonates, so we could not include them in the study. Otherwise, the cohort would have been more extensive. Secondly, the study was a single-centered study and cannot be generalized. Each center should have its own reference level for the population that it caters.

The study's strength is that it is the first to depict the reference cutoff for all seven parameters of NBS from this part of Central India. The laboratory followed a stringent quality control check and robust individual-level data collection. Published articles regarding the reference cutoff are few from developing countries like India, where LC/MS-MS facilities are scarce. However, detailed clinical data of the babies by following them for up to one year would be appropriate enough to provide sufficient insight regarding the cutoff levels. Most of the time, these milder forms of a disorder might not be identified even by LC/MS-MS. Therefore, considering the clinical scenario of the infant might not be ignored.

## Conclusions

The 99th percentile values were considered as the new cutoff for nTSH, n17-OHP, nPKU, nIRT, and nGAL. For nG6PD and nBIOT, lower percentile, 1.0th percentile values were accounted as the new cutoff levels. The n17-OHP and nIRT levels significantly differed among the infants of the first five days indicating that it might take a few days to achieve a baseline value. To be more precise, the MoM value may also be reported along with the result of the parameter. It is therefore important to understand the dynamics of the parameters and accordingly define the reference cutoff level in the local population for timely evaluation of the health in neonates. Large-scale longitudinal studies with biochemical analysis in LC/MS-MS and a complete analysis of determinants influencing the NBS parameters should be considered to define the cutoff values precisely.
